# Real-world applicability of glial fibrillary acidic protein and neurofilament light chain in Alzheimer’s disease

**DOI:** 10.3389/fnagi.2022.887498

**Published:** 2022-08-22

**Authors:** Tandis Parvizi, Theresa König, Raphael Wurm, Sara Silvaieh, Patrick Altmann, Sigrid Klotz, Paulus Stefan Rommer, Julia Furtner, Günther Regelsberger, Johann Lehrner, Tatjana Traub-Weidinger, Ellen Gelpi, Elisabeth Stögmann

**Affiliations:** ^1^Department of Neurology, Medical University of Vienna, Vienna, Austria; ^2^Division of Neuropathology and Neurochemistry, Department of Neurology, Medical University of Vienna, Vienna, Austria; ^3^Division of Neuroradiology and Musculoskeletal Radiology, Department of Biomedical Imaging and Image-Guided Therapy, Medical University of Vienna, Vienna, Austria; ^4^Division of Nuclear Medicine, Department of Biomedical Imaging and Image-Guided Therapy, University of Vienna, Vienna, Austria

**Keywords:** Alzheimer’s disease, dementia, biomarker, GFAP, NfL

## Abstract

**Background:** Blood-based biomarkers may add a great benefit in detecting the earliest neuropathological changes in patients with Alzheimer’s disease (AD). We examined the utility of neurofilament light chain (NfL) and glial fibrillary acidic protein (GFAP) regarding clinical diagnosis and differentiation between amyloid positive and negative patients. To evaluate the practical application of these biomarkers in a routine clinical setting, we conducted this study in a heterogeneous memory-clinic population.

**Methods:** We included 167 patients in this retrospective cross-sectional study, 123 patients with an objective cognitive decline [mild cognitive impairment (MCI) due to AD, *n* = 63, and AD-dementia, *n* = 60] and 44 age-matched healthy controls (HC). Cerebrospinal fluid (CSF) and plasma concentrations of NfL and GFAP were measured with single molecule array (SIMOA^®^) technology using the Neurology 2-Plex B kit from Quanterix. To assess the discriminatory potential of different biomarkers, age- and sex-adjusted receiver operating characteristic (ROC) curves were calculated and the area under the curve (AUC) of each model was compared.

**Results:** We constructed a panel combining plasma NfL and GFAP with known AD risk factors (Combination panel: age+sex+*APOE4*+GFAP+NfL). With an AUC of 91.6% (95%CI = 0.85–0.98) for HC vs. AD and 81.7% (95%CI = 0.73–0.90) for HC vs. MCI as well as an AUC of 87.5% (95%CI = 0.73–0.96) in terms of predicting amyloid positivity, this panel showed a promising discriminatory power to differentiate these populations.

**Conclusion:** The combination of plasma GFAP and NfL with well-established risk factors discerns amyloid positive from negative patients and could potentially be applied to identify patients who would benefit from a more invasive assessment of amyloid pathology. In the future, improved prediction of amyloid positivity with a noninvasive test may decrease the number and costs of a more invasive or expensive diagnostic approach.

## Introduction

Alzheimer’s disease (AD) represents a frequent neurodegenerative disorder, which leads to a progressive decline in cognitive functions (McKhann et al., 1984, 2011). Since the earliest neuropathological changes with the cerebral accumulation of amyloid-beta (Aβ) and neurofibrillary tangles (NFT) are expected to begin 10–20 years before clinical manifestation, the definition of AD shifted towards a rather biological construct with a better understanding of AD as a disease continuum (Sperling et al., 2011; Bateman et al., 2012; Jack et al., 2018). The diagnosis of early phases of AD is of particular interest concerning the inclusion in clinical trials and the development of disease-modifying therapies. Recent studies have been looking for a possibility to identify reliable blood-based biomarkers for an early AD diagnosis, as nowadays biomarker diagnosis is either performed with cost-intensive positron emission tomography (PET) imaging or invasive lumbar puncture.

The establishment of new and sensitive analytical methods may facilitate this approach. In comparison to the already established enzyme-linked immunosorbent assay (ELISA), the development of ultrasensitive single molecule arrays (SIMOA^®^) has improved the sensitivity of detecting proteins in the femtomolar range (Barro et al., 2020; Abdelhak et al., 2022).

Neurofilament light chain (NfL), a subunit of specific cytoskeletal proteins of neurons, represents a highly proposed biomarker for the detection of neuronal loss. Cerebrospinal fluid (CSF) and blood NfL levels are increased in the vast majority of neurological conditions with the highest concentrations in individuals with human immunodeficiency virus (HIV)-associated dementia, frontotemporal dementia (FTD), and amyotrophic lateral sclerosis (ALS; Bridel et al., 2019; Ashton et al., 2021). Furthermore, NfL is also elevated in AD and studies on autosomal dominant AD showed an elevation of NfL over a decade before the expected onset of clinical symptoms (Preische et al., 2019). Higher NfL levels are associated with cognitive decline, brain atrophy, and future disease progression in multiple neurological disorders (Mattsson et al., 2017; Lewczuk et al., 2018; Bridel et al., 2019). Additionally, several studies have indicated the use of NfL as a marker for treatment response (Olsson et al., 2019; Delcoigne et al., 2020).

Another promising biomarker for tracking neurodegenerative changes could be glial fibrillary acidic protein (GFAP), an intermediate filament protein of astrocytes. Neuropathological data have shown a close spatial relationship between reactive astrocytes and amyloid plaques in brain tissue of patients with AD (Verkhratsky et al., 2010; Kamphuis et al., 2014). Increased GFAP concentrations have been detected in CSF and blood of AD patients, with rising levels already at the preclinical phase of the disease, as well as an association between GFAP levels and cerebral amyloid pathology, brain atrophy, cognitive decline, and future conversion to dementia (Elahi et al., 2019; Oeckl et al., 2019; Asken et al., 2020; Verberk et al., 2020; Benedet et al., 2021; Chatterjee et al., 2021; Cicognola et al., 2021). Furthermore, an elevation of GFAP has been observed in patients with traumatic brain injury, neuroinflammatory, and other neurodegenerative disorders including Lewy body dementia (LBD) and progranulin-associated FTD (Heller et al., 2020; Katisko et al., 2021; Zhu et al., 2021; Abdelhak et al., 2022; Chouliaras et al., 2022).

The aim of this study was to examine GFAP and NfL levels in CSF and plasma in various stages of the clinical AD continuum and to investigate the predictive value of these blood biomarkers in combination with well-established risk factors in relation to clinical diagnosis and amyloid positivity. Due to the fact, that most biomarker studies include a preselected population with stringent eligibility criteria, we aimed to evaluate the real-world application of these biomarkers in a relatively heterogenous population of memory-clinic outpatients.

## Methods

### Study population

One-hundred sixty-seven patients were enrolled in this retrospective study at the Memory Clinic of the Department of Neurology, Medical University of Vienna (MUV). As various patients with the main concern of subjective/objective cognitive decline are referred to our specialized memory clinic both by specialists and generalists, without a preselection, our patient cohort rather reflects a more heterogeneous study population and thus resembles more closely a real-world setting. Using two existing registries, the Dementia Registry RDA MUV (EK 1323/2018) and the BIOBANK MUV (EK 2195/2016), we identified 123 patients with a diagnosis along the clinical spectrum of cognitive decline, i.e., mild cognitive impairment (MCI, *n* = 63) due to AD and AD-dementia (*n* = 60). Additionally, 44 age-matched healthy controls (HC) were included. These participants were recruited from an unselected population of patients, that were administered to the Department of Neurology and received further neurological examination, including brain imaging and lumbar puncture, to rule out an underlying neurological disorder. The main diagnoses of these patient cohorts consisted of idiopathic cranial nerve palsies, headache syndromes, and somatic symptom disorders and showed no signs of a neurodegenerative disease or subjective/objective cognitive decline.

All 123 patients with an objective cognitive decline (MCI, AD) underwent a thorough standardized diagnostic examination including physical and neurological evaluation, neuropsychological testing, magnetic resonance imaging (MRI) of the brain, and basic laboratory testing. For a subset of patients, we extended our diagnosis with a biomarker-based approach. CSF analysis of established AD biomarkers [amyloid-beta 42 (Aβ42), total tau (tTau), and phosphorylated tau (pTau)] was available in 75 patients, amyloid-PET imaging was performed in 80 patients, and 60 patients underwent both diagnostic methods.

Diagnoses of MCI and dementia due to AD were based on the recommendation of the National Institute of Ageing and Alzheimer’s Association (NIA-AA; Albert et al., 2011; McKhann et al., 2011). All 167 study participants were required to have a plasma EDTA sample stored in the Biobank MUV, for 103 study participants CSF samples were available as well.

The project was approved by the Ethics Committee of the Medical University of Vienna (EK 1965/2019) on November 28th, 2019.

### Neuropsychological assessment

The Neuropsychological Test Battery Vienna (NTBV) was administered to assess cognitive function, including domains of attention, language, executive functioning, and episodic memory (Pusswald et al., 2013; Lehrner et al., 2015a). Adequate normative data from cognitively unimpaired individuals were available and z-scores for each variable were calculated and corrected for age, education, and sex. Screening of cognitive impairment consisted of Mini-Mental State Examination (MMSE), Global Deterioration Scale (GDS), and *Wortschatztest* (WST), a standardized vocabulary test providing an estimate of premorbid intelligence level (Schmidt and Metzler, 1992). Furthermore, the Vienna-Visuo-Constructional Test 3.0 (VVT-3.0) was applied to assess the visuo-constructive performance (Lehrner et al., 2015b). Depressive symptoms were measured *via* Beck Depression Inventory (BDI-II; Kühner et al., 2007).

### APOE genotyping

Apolipoprotein E (*APOE*) genotyping was performed in 143 patients using quantitative polymerase chain reaction (qPCR) with TaqMan probes (Thermofisher) evaluating two single nucleotide polymorphisms (SNPs) in the *APOE* gene (rs429358 and rs7412). Each sample was tested for both SNPs in triplicates using 20 ng deoxyribonucleic acid (DNA). Allelic discrimination analysis was used to determine the *APOE* genotype of the study participants.

### MR imaging

All patients underwent at least a T1-weighted MR sequence, a T2-weighted or a Fluid-attenuated inversion recovery (FLAIR) MR sequence, and a diffusion-weighted MR sequence within the routine diagnostic setting for the evaluation of the extent and pattern of atrophy, the presence and degree of vascular lesions and to exclude other underlying pathologies causing cognitive decline and diffusion restricted areas.

### Amyloid-PET imaging

Eighty patients underwent an amyloid-PET scan with [^18^F] flutemetamol (*n* = 28) or [^11^C] Pittsburgh compound-B (PiB, *n* = 52). Amyloid-PET imaging was performed on one of two possible PET scanner systems (Siemens Biograph 64 True Point, Erlangen, Germany or GE Advances PET, GE Healthcare Institute, Waukesha, Wisconsin, USA). All studies were performed under strictly controlled conditions. In short, either ~400 MBq of [11C] PiB (in-house production according to previously published recommendations; Philippe et al., 2011) or 185 MBq of [18F] flutemetamol (Vizamyl^®^, GE Healthcare) were injected intravenously into a peripheral vein with starting image acquisition 40 min p.i. for [11C] PiB and 90 min p.i. for Vizamyl^®^, where the tracer accumulation in the brain is reaching the maximum. Subsequently, the image acquisition was performed for about 20 min following a computed tomography (CT) acquisition for attenuation correction using Siemens Biograph 64 True Point.

Scans were rated visually as positive or negative for the presence of amyloid pathology in the cortex by an experienced nuclear medicine physician according to the guidelines of the tracer manufacturers.

### Fluid biomarkers

CSF was obtained by lumbar puncture between the L3/L4, L4/5, or L5/S1 intervertebral space, collected in polypropylene tubes and further stored at −20°C until biomarker analysis (as for Aβ42, pTau 181, and tTau), or immediately at −80°C for future research purposes (Teunissen et al., 2014; Duits et al., 2015). Levels of Aβ42, pTau 181, and tTau were measured with commercially available ELISA (Innotest hTAU-Ag, Innotest phosphoTAU 181p, Innotest beta-amyloid 1–42; Vanmechelen et al., 2000; Vanderstichele et al., 2009). The cut-off for these biomarkers were based on the manufacturer’s recommendation (Aβ42 < 500 pg/ml, pTau 181 > 61 pg/ml, tTau > 300 pg/ml). From these measurements, Innotest Amyloid Tau Index (IATI) was calculated for each patient (measured as Aβ42/(240+1.18xtTau), reference values <1 pg/ml indicative of AD pathology, >1 pg/ml—normal; Hulstaert et al., 1999; Tabaraud et al., 2012).

EDTA plasma was collected through venepuncture and stored at −80°C in our local biobank. Concentrations of NfL and GFAP were quantified with an ultrasensitive single molecule array (SIMOA^®^) using the Neurology 2-Plex B kit from Quanterix in CSF and plasma. Detailed analyses are described elsewhere (Altmann et al., 2020). In short, equilibrated calibrators, samples, and controls were diluted (1:4 for plasma and 1:100 for CSF) and incubated with detector and paramagnetic reagents provided by the manufacturer. Streptavidin ß-galactosidase was added to each well before samples were transferred to the Quanterix SR-X analyzer for measurement of protein levels. All samples were analyzed as duplicates and all assay materials were obtained from the same kit lot. Intra-assay coefficient of variation (CV) was <12% for GFAP Plasma, <13% for GFAP CSF, <9% for NfL Plasma and <8% for NfL CSF. Inter-assay CV for two samples measured repeatedly on 10 plates was well acceptable (<12% for GFAP Plasma, <14% GFAP CSF, <8% NfL Plasma and <10% NfL CSF). Five patient samples were excluded from further analysis due to a high CV (>20%) and therefore not included in this study.

### Amyloid positivity

Amyloid positivity was defined by CSF (IATI < 1) and/or amyloid-PET imaging. In cases where both examinations were available or discordant results were obtained, amyloid status was determined by PET.

### Statistical analysis

Data are presented as n (percent) or median (interquartile range) as appropriate. Testing for differences between groups was performed using the chi-square test, the Mann-Whitney-U-test, or the Kruskal-Wallis-test. The correlation was assessed using Spearman’s rank correlation coefficient. To evaluate the discriminatory performance of the biomarkers assessed herein, the cohort was split into pairs of two diagnoses (e.g., AD and HC) and the response variable was coded as existing for the more severe diagnosis (i.e., MCI when assessing MCI vs. HC). Next, a baseline model consisting of sex, age, and *APOE4* status was constructed using logistic regression. A receiver operating characteristic (ROC) curve was plotted and the area under the curve (AUC) was measured. Optimal cutoffs were calculated using Youden’s J-Statistic (Youden, 1950). The baseline model was then supplemented by either level of plasma GFAP, plasma NfL, or both, and the AUC of each model was compared using DeLong’s test for correlated AUC curves (DeLong et al., 1988). A *p*-value of <0.05 was interpreted as statistically significant. All calculations were performed in R (Version 4.0.4) and the pROC package was used for ROC calculations (Robin et al., 2011).

## Results

### Participant characteristics

Demographic and clinical characteristics are listed in Table 1.

**Table 1:** Demographics and clinical characteristics.

	**HC (*n* = 44)**	**MCI (*n* = 63)**	**AD (*n* = 60)**	***p* value**
Sex (f)	24 (54.5%)	29 (46%)	36 (60%)	*p* = 0.294
Age	61.2 (55.8, 69.5)	69.9 (59.3, 77.8)	69 (61.3, 75)	*p* < 0.01
MMSE	n.a.	27 (25, 28)	20 (14, 23)	*p* < 0.001
*APOE4* carrier n/ total n (%)	12/36 (33.3%)	22/54 (40.7%)	33/53 (62.3%)	*p* < 0.05
CSF Aβ42 (pg/ml)^*^	n.a.	354 (248, 479.5)	332.5 (231.8, 454.8)	*p* = 0.322
CSF tTau (pg/ml)^*^	n.a.	310 (188, 504.5)	600.5 (404.3, 1106.8)	*p* < 0.001
CSF pTau (pg/ml)^*^	n.a.	53 (33.5, 79.5)	77.5 (51.3, 96.3)	*p* < 0.05
CSF IATI (pg/ml)^*^	n.a.	0.6 (0.3, 0.8)	0.3 (0.2, 0.5)	*p* < 0.001
Amyloid-PET positivity n/total n (%)	n.a.	23/39 (59%)	39/41 (95.1%)	*p* < 0.001
Amyloid positivity n/total n (%)^**^	n.a.	29/46 (63%)	47/49 (95.9%)	*p* < 0.001
Plasma NfL (pg/ml)	8.1 (5.9, 12.2)	12. 9 (8.5, 20.4)	15.5 (11.8, 23.2)	*p* < 0.001
Plasma GFAP (pg/ml)	79 (53.7, 120.6)	167.5 (93.8, 256.3)	181.9 (129.6, 269.6)	*p* < 0.001
CSF NfL (pg/ml)^***^	584.1 (449.6, 832.8)	807.7 (507.7, 1103.2)	1,559 (1026.6, 2513.9)	*p* < 0.001
CSF GFAP (pg/ml)^***^	11,145.3 (6980.5, 14373.8)	8,946.2 (7028.8, 13842.7)	13,663.5 (9945.4, 21059.1)	*p* < 0.01

Data are presented as the median and interquartile range (IQR, 25th–75th percentile) or n (%). Demographic and clinical differences were measured using the Kruskal-Wallis test or the chi-square tests as appropriate. ^*^CSF AD biomarkers (Aβ42, tTau, pTau, IATI) were available for 75 patients (37 MCI, 38 AD). ^**^Amyloid positivity was defined by CSF IATI <1 pg/ml and/or positive amyloid-PET imaging. ^***^CSF NfL and GFAP levels were analyzed in 103 patients (36 HC, 30 MCI, 37 AD). HC, healthy controls; *MCI*, mild cognitive impairment; *AD*, Alzheimer’s disease; *f*, female; *MMSE*, mini-mental state examination; *CSF*, cerebrospinal fluid; *Aβ42*, amyloid-beta 42; *tTau*, total tau; *pTau*, phosphorylated tau; *IATI*, Innotest Amyloid Tau Index; *NfL*, neurofilament light chain; *GFAP*, glial fibrillary acidic protein; *n.a*., not available; *NfL*, neurofilament light chain; *GFAP*, glial fibrillary acidic protein.

We observed no significant difference in sex distribution between the groups, while HC were significantly younger than the two patient groups (*p* < 0.01 for HC vs. MCI and AD). MMSE decreased significantly with progressing disease with the lowest score in the AD group (*p* < 0.001). Data of *APOE4* carriership (carriers of at least one *APOE4* allele) was available for 143 patients, with the highest occurrence of *APOE4* alleles in AD patients (33 of 53 patients, 62.3%), compared to 22 out of 54 patients in the MCI group (40.7%). A chi-square test of independence was performed to examine the relationship between the *APOE4* status and the diagnosis. As can be seen by the frequencies cross-tabulated in Table 1, there was a significant relationship between *APOE4* status and diagnosis (X^2^_(2, *N* = 167)_ = 8.5078, *p* < 0.05.

For a subset of patients (*n* = 75) CSF analysis of established AD biomarkers was available (Aβ42, tTau, pTau). While CSF tTau and pTau levels increased significantly with progression from MCI to AD (*p* < 0.001 and *p* < 0.05, respectively), the difference in Aβ42 concentration between MCI and AD reached no statistical significance. Accordingly, the IATI value was significantly lower in the AD group than in the MCI group (*p* < 0.001).

Amyloid-PET imaging was performed in 39 of 63 patients with MCI (61.9%) and 41 of 60 patients with AD (68.3%). Positive amyloid-PET imaging was significantly higher in AD patients with a total of 39 (95.1%) positive subjects in AD, compared to 23 patients with MCI (59%, *p* < 0.001). Taken together, biomarker data (CSF analysis or PET imaging) was available for 95 patients (56.9%), which demonstrated signs of amyloid pathology in a total of 76 patients (80%), determined by CSF IATI and/or amyloid-PET imaging as outlined previously.

### Concentration of GFAP and NfL in plasma and CSF

**Figure 1:**
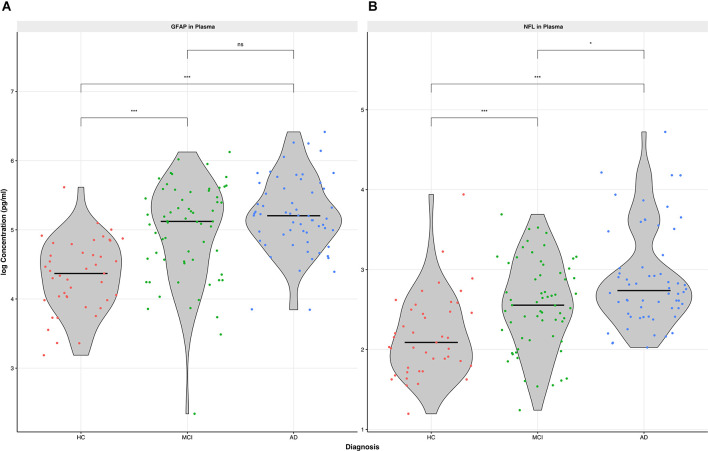
Concentration of GFAP **(A)** and NfL **(B)** in plasma among the three cohorts (HC, MCI, AD). Differences of biomarker concentration were calculated using Kruskal-Wallis Test, p value is displayed as *p < 0.05, ***p < 0.001, *ns*, not significant. *HC*, healthy controls; *MCI*, mild cognitiveimpairment; *AD*, Alzheimer’s disease; *CSF*, cerebrospinal fluid; *NfL*, neurofilament light chain; *GFAP*, glial fibrillary acidic protein.

Plasma GFAP displayed a gradual increase along the three cohorts, with the highest concentration in patients with AD (median 181.9 pg/ml, IQR 129.6, 269.6, Table 1 and Figure 1A). While plasma levels were significantly higher in patients with MCI vs. HC and AD vs. HC (*p* < 0.001), we observed no significant difference of plasma GFAP levels between MCI vs. AD.

Plasma NfL performed similarly to GFAP regarding the difference in concentrations between MCI vs. HC and AD vs. HC (*p* < 0.001, Table 1 and Figure 1B). In contrast to plasma GFAP, NfL levels showed a significant discrimination between MCI vs. AD (*p* < 0.05).

For 103 patients, CSF samples in our local biobank were available. Levels of CSF NfL increased gradually, with the lowest concentration in the HC group (median 584.1 pg/ml, IQR 449.6, 832.8) and the highest concentration in the AD group (median 1,559 pg/ml, IQR 1,026.6, 2,513.9).

On the contrary, CSF GFAP presented the lowest concentration in the MCI group (median 8,946.2 pg/ml, IQR 7,028.8, 13,842.7), followed by HC (median 11,145.3 pg/ml, IQR 6,980.5, 14,373.8) and AD (median 13,663.5 pg/ml, IQR 9,945.4, 21,059.1).

Both CSF biomarker levels allowed a good distinction between AD vs. HC and MCI vs. AD (NfL *p* < 0.001 for both measurements, GFAP *p* < 0.05 and *p* < 0.01, respectively), while a significant differentiation between HC vs. MCI could not be demonstrated.

In a logistic regression model including GFAP or NfL as the dependent variable and age and diagnosis (with HC as the comparator) as predictors, age was significantly associated with GFAP (*B* = 4.2, *p* < 0.001) and NfL (*B* = 0.4, *p* < 0.001), while diagnosis remained significant in both models.

**Figure 2:**
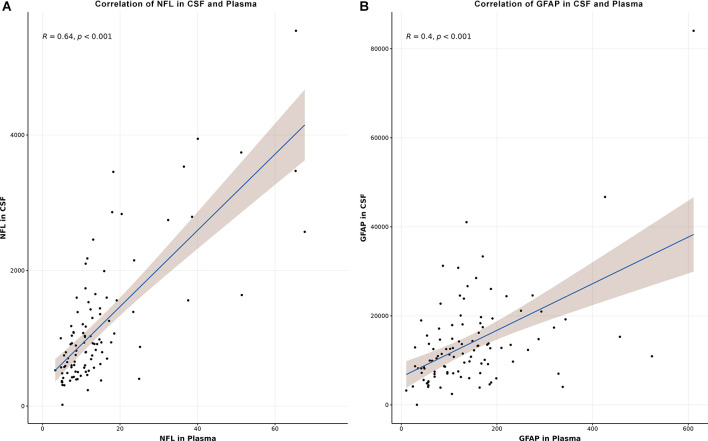
Correlation of NfL in CSF and plasma **(A)** and GFAP in CSF and plasma**(B)**. Correlation was assessed using Spearman’s rank correlation coefficient. *CSF*, cerebrospinal fluid;*NfL*, neurofilament light chain; *GFAP*, glial fibrillary acidic protein.

Using Spearman correlation coefficient, the correlation of NfL and GFAP in CSF and plasma were analyzed (Figures 2A,B). Correlation between NfL in CSF and plasma performed better (*R* = 0.64, *p* < 0.001, Figure 2A) than the correlation of GFAP in CSF and plasma (*R* = 0.4, *p* < 0.001, Figure 2B).

### Diagnostic value of plasma GFAP and NfL in combination with known AD risk factors

**Figure 3:**
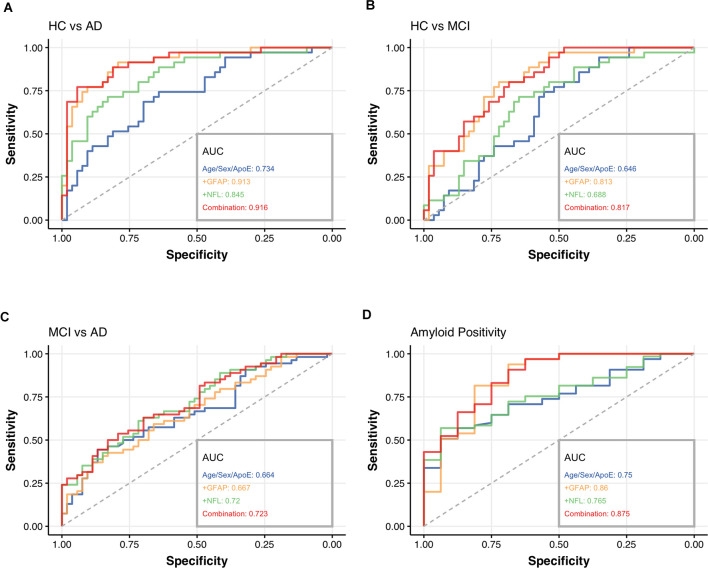
Receiver operating characteristic (ROC) curves for the diagnostic performance in distinguishing HC from AD **(A)**, HC from MCI **(B)**, MCI from AD **(C)**, the differentiation between amyloid positive and negative patients in our cohort **(D)**. The area under the curve (AUC) of each model was compared using DeLong’s test for correlated AUC curves. The four panels analyzed were called age+sex+*APOE4* (blue), GFAP+ (age+sex+*APOE4*+GFAP, orange), NfL+ (age+sex+*APOE4*+NfL, green), and Combination panel (age+sex+*APOE4*+GFAF+NfL, red) in this figure. *HC*, healthy controls; *MCI*, mild cognitive impairment; *AD*, Alzheimer’s disease; *NfL*, neurofilament light chain; *GFAP*, glial fibrillary acidic protein; *AUC*, area under the curve.

To assess the clinical utility of GFAP and NfL in plasma, particularly in distinguishing healthy controls from patients with cognitive complaints (MCI and AD) and potentially predicting cerebral amyloid status, ROC analyses were performed and adjusted for sex and age. We constructed a diagnostic panel, consisting of well-established risk factors such as age, sex (defined as female > male), and *APOE4* carriership (defined as carrying at least one copy of the *APOE4* allele; i.e., age+sex+*APOE4* panel) and compared it with a panel of age, sex, *APOE4* carriership added by plasma NfL and plasma GFAP, called combination panel (i.e., age+sex+*APOE4*+GFAP+NfL panel, Figures 3A–D). Additionally, we analyzed each biomarker separately to evaluate the potential benefit of GFAP or NfL alone (i.e., age+sex+*APOE4*+GFAP panel and age+sex+*APOE4*+NfL panel).

When using the age+sex+*APOE4* panel alone, we calculated an AUC of 73.4% (95%CI = 0.63–0.84) for HC vs. AD (Figure 3A), AUC of 64.6% (95% CI = 0.53–0.76) for HC vs. MCI (Figure 3B) and an AUC of 66.4% (95%CI = 0.56–0.77) for MCI vs. AD (Figure 3C). Regarding the diagnostic accuracy in predicting amyloid status and the distinction of amyloid-negative (Aβ-) from amyloid-positive (Aβ+) individuals, the AUC was 75% (95% CI = 0.62–0.88, Figure 3D).

By adding NfL to the panel (age+sex+*APOE4*+NfL panel), the discrimination between HC vs. AD reached a significantly higher AUC of 84.5% (95%CI = 0.76–0.93, Figure 3A) compared to the age+sex+*APOE4* panel alone (*p* = 0.003), while the other calculations failed to achieve significantly better results (HC vs. MCI AUC 68.8%, 95%CI = 0.58–0.80, MCI vs. AD AUC 72%, 95%CI = 0.63–0.82), amyloid positivity (AUC 76.5%, 95%CI = 0.61–0.87, Figures 3B–D).

The combination of GFAP with the age+sex+*APOE4* panel (age+sex+*APOE4*+GFAP panel) obtained an AUC of 91.3% (95%CI = 0.85–0.97) for HC vs. AD (*p* < 0.001, Figure 3A) and AUC of 81.3% (95% CI = 0.72–0.90) for HC vs. MCI (*p* < 0.01, Figure 3B) compared to the age+sex+*APOE4* panel. The prediction of amyloid positivity demonstrated an AUC of 86% (95%CI = 0.70–0.97, Figure 3D), but missed statistical significance as well as the differentiation between MCI vs. AD (AUC 66.7%, 95%CI = 0.57–0.77, Figure 3C).

When combining the two biomarkers with the age+sex+*APOE4* panel (combination panel: age+sex+*APOE4*+GFAP+NfL), the AUC of HC vs. AD reached 91.6% (95%CI = 0.85–0.98, *p* < 0.001, Figure 3A), AUC of HC vs. MCI 81.7% (95%CI = 0.73–0.90, *p* < 0.01, Figure 3B) and for amyloid positivity 87.5% (95%CI = 0.73–0.96, *p* < 0.05, Figure 3D), therefore, significantly outperforming the age+sex+*APOE4* panel alone. Similar to the other two panels (age+sex+*APOE4*+GFAP panel and age+sex+*APOE4*+NfL panel), the combination panel could not improve the distinction between MCI vs. AD (AUC 72.3%, 95%CI = 0.63–0.82, Figure 3C).

## Discussion

In this outpatient memory clinic-based study, we examined the performance of two promising biomarkers of neurodegeneration and neuroinflammation, NfL, and GFAP, for the diagnostic work-up of patients along the continuum of AD-related cognitive decline. We aimed to develop a practical and reproducible model for a quick and accurate patient at-risk identification in a routine clinical practice.

A combination of demographic factors with *APOE*4 status and blood biomarkers, such as GFAP and NfL, might offer a reliable differentiation between healthy controls and patients with an objective cognitive decline, particularly between healthy controls and patients with AD. In terms of predicting amyloid positivity in a cognitively impaired cohort, an integrated approach of history and blood analysis could also serve as a feasible and accessible tool, especially in screening those patients, who might need a more detailed and effortful diagnostic approach. By additional assessment of these two plasma biomarkers, the diagnostic accuracy as well as the prediction of cerebral amyloid accumulation could be majorly improved. Interestingly, this effect was more pronounced for plasma GFAP than plasma NfL. This could be explained by the fact, that GFAP seems to be a marker of the earliest AD pathology with an association between GFAP levels and amyloid load, while NfL could be more useful in terms of disease monitoring and progression (Verberk et al., 2021; Ebenau et al., 2022). Unfortunately, for discriminating the disease states of MCI and AD, none of the three biomarker-based panels could add a significant benefit to the age+sex+*APOE4* panel. However, due to the better understanding of AD as a continuum, this clear distinction is getting more and more ambiguous (Jack et al., 2018).

Focusing on plasma GFAP alone, its levels showed a gradual increase along the three cohorts, with the highest concentration in patients with AD, thereby allowing a good biological interpretation of a gradual rise of this biomarker along the progressing neuropathological process. The most prominent discrimination was achieved between HC and patients with an objective cognitive decline (MCI and AD). However, in contrast to NfL alone, plasma GFAP could not differentiate between MCI vs. AD.

Regarding CSF biomarkers in our cohort, we found the highest concentration of CSF GFAP in patients with AD followed by the HC group and the lowest concentration in MCI, therefore, these results must be interpreted cautiously. CSF NfL demonstrated a gradual increase over the three cohorts with the lowest levels in HC and the highest in AD. Nevertheless, both measurements—CSF GFAP and CSF NfL—allowed a good discrimination between HC and AD as well as MCI and AD, with better results for CSF NfL. The concentration of NfL in CSF and plasma correlated well with each other, which is in line with already published data (Kuhle et al., 2016; Rojas et al., 2016; Mattsson et al., 2017), suggesting that plasma levels might be considered as an acceptable proxy for CSF levels. In contrast to NfL, levels of GFAP in CSF and plasma showed a lower correlation, which was already described by another study (Oeckl et al., 2019). Recently published data indicated higher effect sizes of the increase of plasma GFAP compared to CSF GFAP and a more accurate distinction between Aβ+ and Aβ- individuals for plasma GFAP. Potential explanations could be preanalytical factors or different clearance mechanisms regarding the disrupted blood-brain barrier in patients with AD (Benedet et al., 2021). Thus, further investigations are needed to better determine the role of CSF GFAP and its correlation with GFAP levels in blood in these patient cohorts.

Diagnosis of the early phases of AD is crucial in regard to detecting patients at risk as early as possible in the development of the neuropathological cascade. Furthermore, due to the limited resources of *in vivo* biomarker testing in the general population, the establishment of a screening tool to select those patients, who would benefit from a more thorough testing, is of great importance. Besides the abnormal aggregation of Aβ peptide and tau protein, neuroinflammation and neurodegeneration represent major components in the pathophysiology of AD (Jack et al., 2018). In recent years, the role of neuroinflammation in the pathogenesis of AD has been increasingly focused on in the literature. Neuropathological data have shown a close spatial relationship between Aβ plaques and reactive astrocytes, which along with microglia, may trigger a pro-inflammatory cascade and eventually lead to neurodegeneration, which in turn activates astrocytes and microglia (Frost and Li, 2017; Garwood et al., 2017). As a cytoskeletal component of astrocytes, GFAP could serve as a promising biomarker reflecting astrocytic activation and proliferation during the neurodegenerative processes, including AD, particularly in its earliest stages (Chatterjee et al., 2021; Verberk et al., 2021). On the other hand, NfL represents a rather unspecific biomarker for neurodegeneration, as it is released by axonal damage in multiple neurological disorders (Forgrave et al., 2019; Thebault et al., 2020). While the importance of NfL as a blood-based biomarker has been already reported in several studies (Mattsson et al., 2017, 2019; Benedet et al., 2019), the significance of GFAP is currently still evolving. To our knowledge, only a few studies have evaluated the combination of GFAP with other biomarkers so far and presented the utility of plasma GFAP not just in discriminating healthy controls from patients with AD but also in distinguishing Aβ+ from Aβ- individuals (Oeckl et al., 2019; Asken et al., 2020; Verberk et al., 2020). Furthermore, higher GFAP levels have been associated with an increased risk for future progression to dementia and a steeper cognitive decline (Cicognola et al., 2021; Verberk et al., 2021).

Regarding the heterogeneity of AD pathology, a panel of well-combined blood-based biomarkers could aid in early detection as well as disease monitoring in the future. Emerging data have proposed plasma-derived pTau 181 and pTau 217 as highly specific biomarkers for AD pathology, which are currently investigated in ongoing studies (Moscoso et al., 2020; Rodriguez et al., 2020; Thijssen et al., 2021). Additional biomarkers, such as GFAP and NfL, could on one side potentially aid in detecting these patients at risk early in the neuropathological cascade and on the other side give further information about disease progression.

While our study population is rather heterogeneous, i.e., more closely resembles a real-world setting, where some patients will not undergo biomarker testing for various reasons, we believe that this adds to the existing literature and confirms the practical usefulness of these biomarkers. As patients undergo a detailed history taking, neurological examination, and blood sampling at the first patient visit, the collection of plasma samples for further biomarker analysis may be easily implemented. Since *APOE* genotyping can be derived from these blood samples and performed in-house in a quick and inexpensive manner, we have added this marker to our proposed panel. In a routine memory clinical setting, the analysis of a panel of blood-derived markers in combination with known risk factors could be of great value concerning the identification of those patients at risk who would need further biomarker testing. This approach could further substantially reduce the number of patients who would otherwise undergo expensive PET imaging or invasive lumbar puncture.

## Limitations

Due to the retrospective nature of this study, established AD biomarkers were not available for the whole study cohort. Positive amyloid status and *APOE4* carriership were significantly less common in the MCI group, which might lead to the notion, that at least some of the MCI patients were not along the AD continuum. Since the patients in the HC group were enrolled based on their clinical performance, we cannot exclude that some of these patients had an underlying AD pathology. Healthy controls were significantly younger than the patient groups, which might influence the results of these biomarkers and their corresponding analysis. To counteract this potential bias in our data, ROC analyses were adjusted for sex and age.

## Conclusion

Blood-based biomarkers for AD may represent a valuable complementary tool for clinical diagnosis and patient management in the near future. We suggest that plasma GFAP could aid in a better distinction of patients along different predementia stages and that the combination of GFAP and NfL plasma levels with conventional risk factors could serve as a good “at-risk” model for selecting those patients, who might need a more invasive or expensive diagnostic approach.

## Data Availability Statement

The raw data supporting the conclusions of this article will be made available by the authors, without undue reservation.

## Ethics Statement

The studies involving human participants were reviewed and approved by Ethics Committee of the Medical University of Vienna. Written informed consent for participation was not required for this study in accordance with the national legislation and the institutional requirements.

## Author Contributions

TP, RW, and ES devised the protocol. TP collected and managed the data with contribution of RW, SS, and ES. TP and TK performed SIMOA analyses in CSF and plasma. TP and RW performed the statistical analysis. TP and ES interpreted the data and prepared the manuscript. RW, TK, PR, SK, EG, PA, TT-W, and JF provided feedback and major contribution to the manuscript. All authors contributed to the article and approved the submitted version.

## Abbreviations

Aβ: Amyloid-Beta
AD: Alzheimer’s disease
ALS: Amyotrophic lateral sclerosis
APOE: Apolipoprotein E
AUC: Area under the curve
BDI-II: Beck Depression Inventory
CSF: Cerebrospinal fluid
CT: Computed tomography
CV: Coefficient of variation
DNA: Deoxyribonucleic acid
ELISA: Enzyme-linked immunosorbent
FLAIR: Fluid-attenuated inversion recovery
FTD: Frontotemporal dementia
GDS: Global Deterioration Scale
GFAP: Glial Fibrillary Acidic Protein
HC: Healthy controls
HIV: Human immunodeficiency virus
IATI: Innotest Amyloid Tau Index
IQR: Interquartile range
LBD: Lewy body dementia
MCI: Mild cognitive impairment
MMSE: Mini-Mental State Examination
MRI: Magnetic resonance imaging
MUV: Medical University of Vienna
NfL: Neurofilament light chain
NTBV: Neuropsychological Test Battery Vienna
NFT: Neurofibrillary tangles
NIA-AA: National Institute on Aging and Alzheimer’s Association
PET: Positron emission tomography
PiB: Pittsburgh Compound B
pTau: Phosphorylated Tau
qPCR: Quantitative polymerase chain reaction
RDA: Research Documentation and Analysis
ROC: Receiver operating characteristic
SCD: Subjective cognitive decline
SIMOA: Single-molecule array
SNP: Single nucleotide polymorphisms
tTau: Total Tau
VVT-3.0: Vienna-Visuo-Constructional Test 3.0
WST: Wortschatztest.
